# Canine fetus immune system at late development

**DOI:** 10.21451/1984-3143-AR2019-0004

**Published:** 2019-10-23

**Authors:** Kelly Cristine Santos Roballo, Aline Fernanda Souza, Valéria Maria Lara, Alessandra Oliveira Pinheiro, Ingrid da Silva Gomes, Rafael Garcia Karam, Daniele dos Santos Martins, Luciana Cristina Machado, Carlos Eduardo Ambrósio

**Affiliations:** 1 Department of Veterinary Medicine, Faculty of Animal Sciences and Food Engineering, University of Sao Paulo, Pirassununga, SP, Brazil.; 2 Department of Surgery, Faculty of Veterinary Medicine and Animal Sciences, University of Sao Paulo, Sao Paulo, SP, Brazil.

**Keywords:** Development, organs, neonate, lymphocytes

## Abstract

The immune system is mainly responsible for protecting the organism against agents that may interfere in its homeostasis. Thus, understand how this system develops and operates is very important, for create new therapies to assist this system in its operation, such as its failure. In domestic dogs, few studies show how actually occurs the development, maturation and functioning of the immune system. Therefore, this study demonstrates the development and possible activation of it on dog fetus from late gestational period by *in situ* and microscopic analyzes.

## Introduction

Embryonic development is the study of early life and its changes through the prenatal period (Moore and Persaud, 2008). The study of development can be divided according to the changes that occur during this period, such as the embryonic stage when most organs and systems are established and the fetal period, which is the growth and improvement of organs (Hyttel *et al*., 2010; Martins *et al*., 2011), thus, the characterization of the embryonic and fetal development is divided according to the changes that occurred during that period (Miglino *et al*., 2006; Pretzer, 2008).

Dog’s immune system still not well described, but in general, it consists of an intricate network of molecules, cells and organs that are responsible for protecting the body against mostly microorganisms, tumors, and traumas (Miglino *et al*., 2006; Tizard, 2014). Furthermore, specialized cells such as macrophages, neutrophils, dendritic cells, natural killer cells, lymphocytes, and components produced by them are part of this system, which is divide in innate and acquire immunity (Hyttel et al., 2010; Abbas et al., 2014).

Innate immunity is formed during embryo and fetus development, in contrast the acquire immunity is only complete at postnatal, due to the evolution of the lymphocyte cell linage and improvement of the antigen recognition after birth (Hyttel *et al*., 2010). The thymus, bone marrow and Peyer’s patch are primary lymphoid organs regulating the initial development and maturation of the immune cells (Hyttel *et al*., 2010).

The canine development happens during approximately 65 days, but the organogenesis starts at 21days (Pieri *et al*., 2015). The immune system begins with the formation of thymus from the third pair of pharyngeal pouches and subsequent formation of secondary lymphoid organs, continuing in the postnatal period (Hyttel *et al*., 2010).

The lymphatic and cardiovascular systems are critical to the immune system, once they lead antigens and antigen presenting cells to the lymph nodes and initiate the immune response, then, entire immune action depends of them (Alitalo and Carmeliet, 2002). In canines, as in cats, around 17 days, the cardiovascular system is formed (Abreu *et al*., 2011; Roballo *et al*., 2013). In addition, the fetal liver represents the initial hematopoietic tissue in early gestation and is responsible for the rapid expansion of restricted progenitor cells lineage from the immune system (Holsapple *et al*., 2003), which can be visualized at around 26 to 27 days of gestation (Pieri *et al*., 2015).

In dogs, the immune system development still unclear and deeper researches are necessary. Consequently, in this study, we analyzed the anatomy and histology of the main immune system organs at late gestation period in domestic dogs (45 to 50 days of pregnancy), to a better understanding of it and future contributions to the immunology studies.

## Materials and Methods

The protocols and procedures involving animals were conducted in accordance with the Committee of Ethics of the Faculty of Veterinary Medicine and Animal Science, University of Sao Paulo, Brazil. Fetus at 45 to 50 days of pregnancy (N = 5, 13.5-15 cm of crown-rump), were obtained from dog population control campaign in Pirassununga, SP, Brazil. In order to define the gestational ages more accurately, we used the methodologies already described by Evans and Sack (1973), Yeager and Concannon (1995), with the aid of a caliper measuring CR length, morphogenesis and organogenesis as well as microscopic techniques. The fetus’s organs were prepared for macroscopy and microscopy analyzes, following Pieri et al. (2015)’s protocol. In summary, they were analyzed *in situ*, and following by fixation for 24h, dehydrated, embedded in paraffin, and serial sections were stained with haematoxylin and eosin, then the slides were analyzed using a Zeiss KS400 (Oberkochen, Germany), and images were captured with AXIVISION 4.6 software (Carl Zeiss, Gottingen, Germany).

## Results

**Figure 1:**
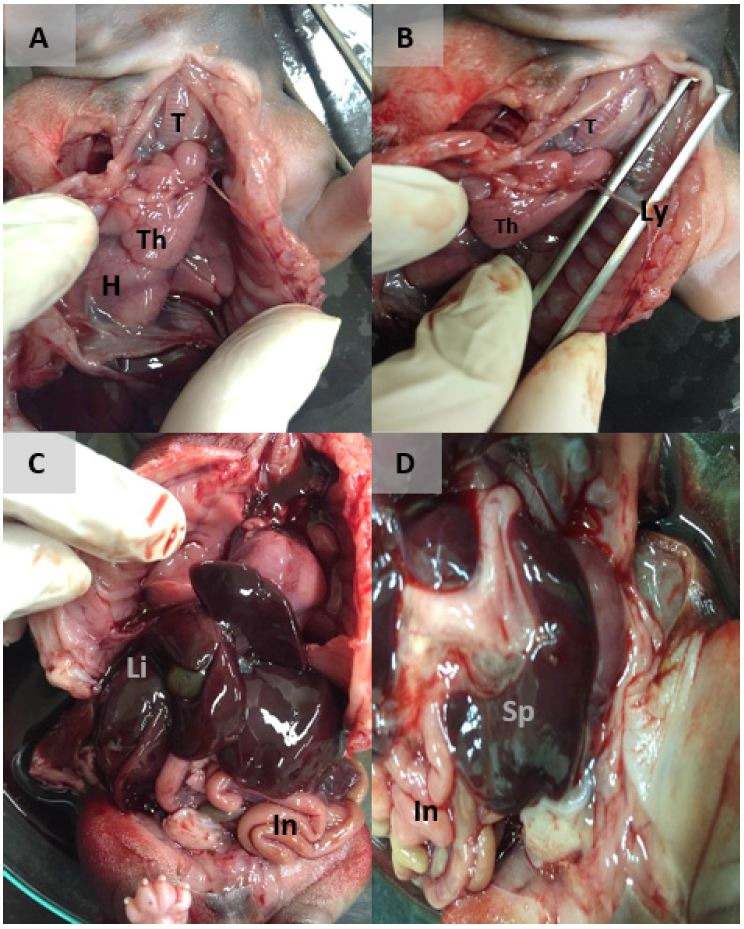
Immune system development in dogs (macroscopy): Trachea (T); Thymus (Th); Heart (H); Lymphocytes (Ly); Liver (Li); Intestine (In) Spleen (Sp).

**Figure 2:**
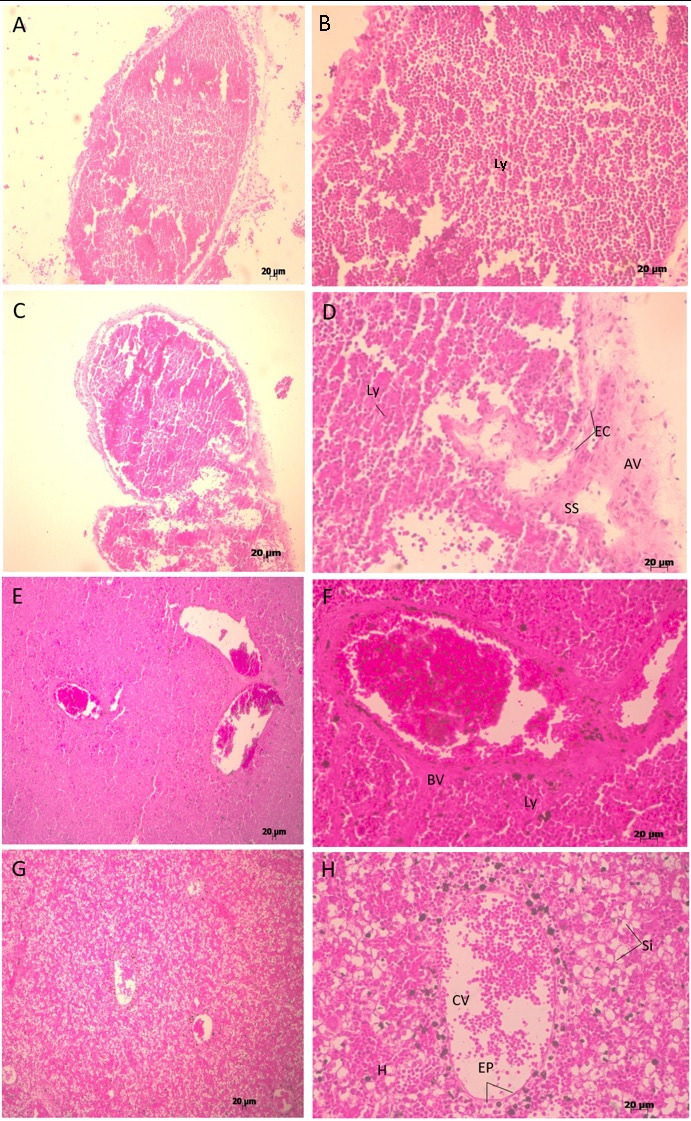
Immune system development in dogs (microscopy): Afferent lymph vessels (AV); subcapsular sinus (SS); Endothelial cells (EC); Lymphocytes (Ly). Central vein (CV); simple squamous epithelium (EP); Hepatocytes (H); Sinusoids (Si). Blood vessels (BV).

The thymus was below the neck, cranial to the heart, close to the aorta artery, in front of the trachea, connected with the main vein and artery from the circulatory system, lobulated and proximally 1.3 mm of length (Fig. 1A and B). In the thymus the medullar and cortical region was not well delimited, the Hassal corpuscles was not evident at this period, and there was apparently reticular cells (important for the lymphocyte’s maturation) (Fig. 2AB).

The lymph node was connected and close to the thymus with less than 0.4 mm of length with afferent lymph vessels, subcapsular sinus, endothelial cells, and lymphocytes, there was no evidence of cortex and medullar region at this fetal period (Fig. 1CD). Moreover, it was possible to analyze this regional lymph node just at this period.

The fetal liver was lobulated, with gallbladder, and occupying about half of the intestinal cavity (Fig. 1CD), and there was apparent central vein formed with blood cells inside with the vein epithelium around and hepatocytes in contact (Fig. 2GH).

The fetal spleen was connected to the circulatory and intestinal systems located at the left side, caudal to the stomach (Fig. 1D), and with lymphocytes, there was not the delimitation of write and red pulp at this period, but the capsule was formed with the simple epithelium (Fig. 2EF).

**Figure 3:**
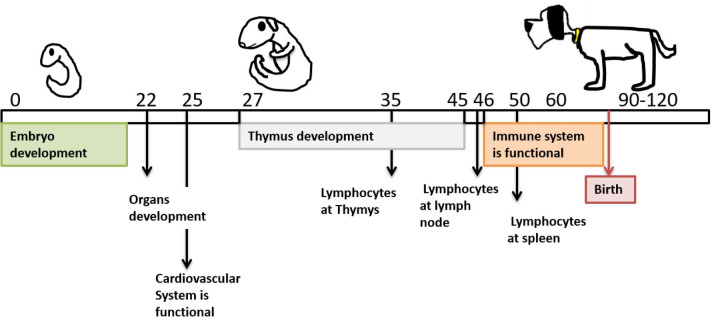
Scheme of the immune system development: In dogs after embryogenesis the cardiovascular system becomes functional allowing the immune system organs maturation (middle third of gestation), and activation (final third of gestation).

In this study, the lymphocytes were present at all main immune system organs at late pregnancy (Fig. 3).

## Discussion

The thymus cellularity in the canine fetal happens at days 45- 55 according to Felsburg (2002) and in accordance with ours findings.

According to Abreu *et al*. (2011), in embryos of cats with approximately 52 days of gestation, the liver occupies a large part of the abdominal cavity and is slightly lobed, with presence of bile duct entering the hepatic parenchyma near the lobular center veins.

Our findings at late gestational period showed the fetal liver without lymphocytes, proving their migration to the immune system organs, in accordance with the following authors’ researches. Solomon (1970) affirms that at early pregnancy periods the appearance of dog lymphocytes occurs much earlier than in cats; in addition, this system become immunocompetent approximately the same physiological period in rabbit, rat, chicken, and possibly dog at early periods. In humans and mice studies the lymphocyte progenitor cells appears in the fetal liver at early pregnancy and migrate (Holsapple *et al*., 2003).

The rudimentary spleen development starts at 27-28 days, being popularized by immune cells after 52 days (Felsburg, 2002), in accordance with the finds of this research.

It knows that lymphocytes derived from bone marrow migrate to the thymus on day 35 to 46, the lymph nodes and spleen around day 50-55 of pregnancy in canines; however, the fetus will be capable of an immune response only in the final third of gestation (Bryant *et al*., 1973; Hyttel *et al*., 2010).

In conclusion, according to our findings, the main immune organs were at this gestational period formed, and there were lymphocyte cells in the canine main organs at late development in dogs (45 to 50 days of pregnancy), proving the immune system functionality, but not proving its total activation. In addition, these results open a new field for other domestic animals immune system researchers.
